# From episodes to populations: evolutionary explanation requires a constructive epistemology

**DOI:** 10.1007/s12064-026-00485-8

**Published:** 2026-06-26

**Authors:** Dan Adler

**Affiliations:** Emdan Research, New York, USA

**Keywords:** Episodic and constructive regimes, Stability-driven assembly, Differential persistence, Evolutionary explanation, No free telos, Prebiotic evolution, Idealization, Lambda calculus

## Abstract

The sciences divide into those that discover laws and those that reconstruct histories. We argue that this division does not reflect a difference in subject matter, but a difference in *epistemic regime*. Law-based sciences operate under *episodic closure*: systems are idealized so that the outcomes of prior interactions do not alter the rules governing future ones. This *regime-defining idealization* (distinguished from *pragmatic idealization*) underlies the predictive successes of physics, but creates a systematic blind spot for evolutionary dynamics. We formalize this distinction using Stability-Driven Assembly (SDA), a minimal non-equilibrium framework in which differential persistence couples episodes into population-level evolutionary dynamics without genes, replication, or predefined fitness functions. Representing compositional objects as *λ*-calculus terms, we show that episodic science studies isolated *λ*-reductions under fixed rules, while evolutionary science studies populations of *λ*-instantiations whose outputs re-enter the space of operators. The resulting dynamics are self-modifying and irreducibly sequential: each step rewrites the conditions for the next. A four-quadrant taxonomy locates episodic science, evolutionary science, and two commonly conflated intermediate cases: formal possibility and constructive potential, within a single framework. From this analysis we derive the “No Free Telos” constraint: in constructive systems where population feedback reshapes the effective dynamics at each step, the cost of predicting future states cannot in general be reduced below the cost of simulating the generative history. The resulting framework bridges episodic and historical sciences, not by reducing one to the other, but by identifying population-level memory as the structural condition that transforms law-governed episodes into open-ended evolutionary processes.

## Introduction: the boundary of “unreasonable effectiveness”

The apparent divide between the physical and biological sciences is widely treated as a difference in subject matter: physics studies simple, law-governed systems, while biology studies complex, historically contingent ones. We argue that this framing is too restrictive. The divide is not necessarily between physics and biology but between two *epistemic regimes*, and any science can occupy either one. What we call *episodic regimes* idealize systems so that experimental trials are independent, state spaces are fixed in advance, and dynamics are governed by transition rules invariant across instantiations. What we call *constructive regimes* are systems in which the outputs of one interaction become the operators for the next, creating a feedback loop in which history accumulates as structure. Physics typically studies the former, whereas biology necessarily studies the latter. But the distinction is structural, not disciplinary: a chemical system allowed to remember its history would become evolutionary, and a biological system forced to forget would become episodic.

**Fig. 1:**
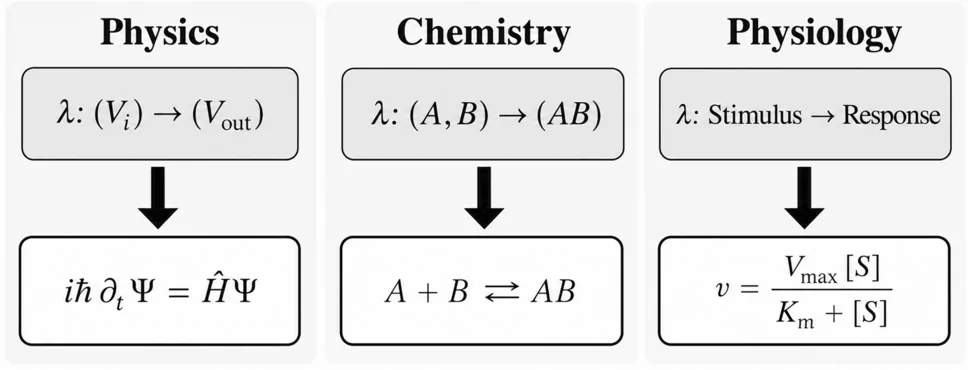
Episodic modeling as *λ*-inference. In physics, chemistry, and physiology, predictive laws (bottom) are induced from discrete observational episodes (top). Each episode is treated as an application of an invariant transformation rule (*λ*-term), discovered by induction over episodes

The “unreasonable effectiveness of mathematics in the physical sciences” (Wigner 1960) may then be a regime selection effect. Mathematics is effective because physical inquiry has historically restricted itself to episodic regimes: fixed phase spaces and deterministic flow in classical mechanics, Markovian approximations in statistical mechanics and chemical kinetics (Van Kampen 2007; Gardiner 2009; Norris 1998), and fixed Hilbert spaces and invariant operator algebras in quantum mechanics (Fig. 1). Under these conditions, inductive inference succeeds: many realizations of an interaction yield a compact causal schema: a closed-form equation or a statistical law that applies universally across contexts (Pearl 2009; Woodward 2003). When mathematics falters in biology, economics, or geology (Cartwright 1983; Mitchell 2003; Garte et al. 2025), the issue may not be a failure of formalism but a mismatch of regime: episodic tools applied to constructive regimes.

**Fig. 2:**
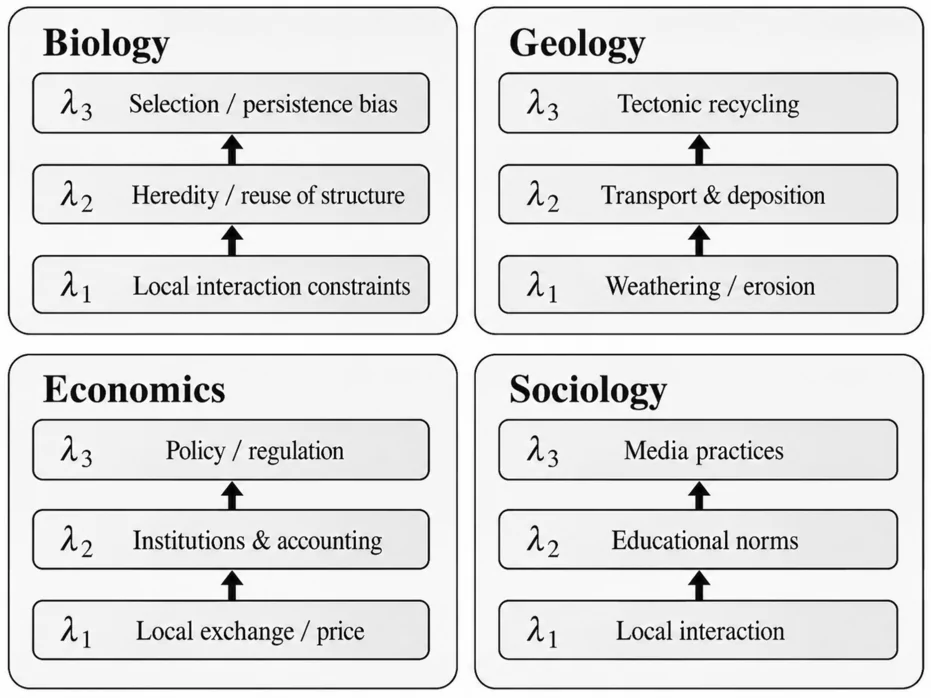
Hierarchical *λ*-inference in historical sciences. In complex domains, the invariant “law” is not a single term but a stack of operators. Each level of constraint ($\lambda _1, \lambda _2, \dots$) emerges from the population dynamics occurring under the constraints of the level below (e.g., stable patterns of local interactions $\lambda _1$ give rise to institutional rules $\lambda _2$). These higher-order, population-level constraints act as shaping contexts for future episodes, creating an evolving system. Labels are illustrative

Constructive regimes, shown schematically in Fig. 2, arise when episodes are allowed to remember each other. The outputs of each *λ*-instantiation do not disappear. They persist, aggregate, and form a population that feeds back to alter the probability distribution of future interactions. The “laws” of the system are no longer fixed invariants, but rather transient constructions of the population history. The framework developed here seeks to explain how such self-modifying dynamics, and the entropy reducing organization they produce, can arise in non-living, open, non-equilibrium systems through stochastic assembly and differential persistence.

We demonstrate this distinction using Stability-Driven Assembly (SDA), a minimal framework in which differential persistence creates the population-level memory required for evolution without genes, replication, or predefined fitness functions. We show that such systems exhibit *generative irreducibility*: the operator governing the system is a *functional* of the population distribution, so that predicting the future requires simulating the constructive history. We term this the “No Free Telos” constraint: there is no general compressed shortcut to the final state because the state space itself is under construction. The resulting framework bridges physics and biology by identifying the epistemic boundary where episodic closure gives way to constructive dynamics.

## Episodic regimes as methodological artifacts

If the distinction between episodic and evolutionary regimes is so fundamental, why has the dependence of the physical sciences on the former not been a detriment to its success? The answer may lie in a specific form of selection bias: the “unreasonable effectiveness” of mathematics in physics is largely the result of restricting inquiry to systems that can be effectively isolated from their histories.

Standard experimental design in physics is an exercise in enforcing Methodological Markovianity. Consider a particle scattering experiment or a chemical reaction study. Great care is taken to ensure that the initial conditions are precisely reset between trials. The apparatus is cleaned; the vacuum is restored; the reactants are purified. By design, the output of $Trial_N$ is prevented from becoming the input to $Trial_{N+1}$. This methodology artificially suppresses the population-level accumulation that characterizes evolutionary systems. It forces the system to behave as a sequence of independent *λ*-episodes, ensuring that the inferred laws are time-invariant and the state space remains fixed.

This methodological stance leads us to distinguish two senses of “memorylessness,” often conflated in scientific practice. *Phenomenological Markovianity* occurs when the system genuinely “forgets” its history due to thermalization or equilibration (e.g., a gas in a box). *Methodological Markovianity* occurs when the system is *prevented* from remembering its history by the intervention of the experimenter (e.g., resetting the apparatus). The former is a property of the target system. The latter is a property of the epistemic framework. When we model evolutionary phenomena using the tools of physics, such as differential equations on fixed manifolds, we are implicitly imposing Methodological Markovianity on systems whose defining feature is the violation of it.

### Regime-defining idealization

Philosophers of science have long recognized the role of idealization in managing complexity. Potochnik argues that idealizations are not merely simplifications but essential tools for isolating causal patterns (Potochnik 2017). We propose a distinction between two fundamentally different types: *Pragmatic idealization* suppresses causal factors that are present but negligible, like ignoring air resistance in a falling body. It acts as a filter, removing noise to reveal a signal, and the suppressed factors can, in principle, be restored without altering the model’s structure. *Regime-defining idealization*, in contrast, structurally eliminates the capacity for state-space expansion, as when a biosphere is modeled as a fixed system of differential equations. This acts not as a filter but as a cage: what is excluded is not a negligible perturbation but the very mechanism by which the system generates novelty.

This distinction refines Cartwright’s influential argument (Cartwright 1983, 1999) that the laws of physics “lie”, holding exactly only within idealized models. For Cartwright, the issue is that real-world complexity prevents the antecedent conditions of laws from being satisfied. In our account, the deeper issue is structural. The distinction is between a world where fixed laws have limited scope and a world where the effective laws are themselves under construction. Standard dynamical models, particularly those intended to capture long-run equilibria, can represent only the former. When we write a system of differential equations $\dot{x} = F(x)$, we pre-specify the dimensionality of the vector *x* and the functional form of *F*. This formalism structurally forbids the emergence of new dimensions (new observables) or the modification of *F* by the system itself. It does not merely simplify the evolutionary process; it eliminates it.

### The “cage” of fixed phase space

This leads to what we term the *Cage Effect*. By modeling a constructive system using episodic tools, we trap the dynamics in a pre-defined phase space. In the episodic regime, a “novelty” is the visitation of a previously unvisited coordinate in a fixed space (a state discovery). In a constructive regime, novelty is the generation of a new coordinate axis altogether (a dimension discovery). Episodic tools can represent the former but structurally preclude the latter. While other accounts have shown that idealizations do constitutive work beyond simplification (Batterman 2002; Morrison 1999), our claim is complementary: regime-defining idealization does not merely filter detail, it cages the dimensionality of what can emerge.

Physics excels at state discovery because its methodology is designed to keep the axes fixed. Biology, geology, and economics struggle under this methodology because their central phenomena involve the proliferation of new axes: new species, new minerals, and new markets. By treating these historical sciences as “messy physics,” we force constructive territories into episodic maps, not merely approximating but structurally mischaracterizing the target phenomenon. We are searching for laws of motion governing the studs, while the phenomenon of interest is the construction of the castle. To model construction, we require a formalism in which composition is not a separate capability, but the engine of dynamics itself. We find this in the lambda calculus.

## The stability-driven assembly (SDA) framework

### Stability-driven assembly: the constructive mechanism

Stability-Driven Assembly (SDA) serves as our operational model for the transition from episodic to constructive dynamics. The framework was introduced by Adler as a minimal non-equilibrium system in which differences in persistence bias the accumulation of structure, with full mathematical derivations demonstrating entropy reduction and scaffold emergence (Adler 2025). In SDA, *stability* and *persistence* denote resistance to dissipation over time, measured as a characteristic lifetime, analogous to half–life in physics. Subsequent work (Adler 2026) instantiated SDA in a chemical symbol space and showed that it behaves as a *natural genetic algorithm* (SDA/GA) with no explicit fitness function: selection arises from differential persistence alone, producing dominant scaffolds and sustained novelty over thousands of generations. For the present argument, we focus on the core dynamical mechanism: how persistence-weighted feedback reshapes the population distribution and induces evolutionary search without explicit replication.

An SDA system consists of a population of interacting entities in an open-flow setting: energy and base elements flow through a bounded interaction volume, maintaining the system far from equilibrium while ensuring that the population is finite and that a well-defined probability distribution over patterns exists at each generation. The dynamics are governed by a simple asymmetry: patterns are generated stochastically but persist differentially. New patterns are created through the interaction of existing ones, e.g., concatenation, bonding, or recombination, depending on the domain. Each pattern is assigned a finite lifetime based on its stability within its domain and surrounding environment. Patterns that persist longer accumulate in the population, increasing their frequency, and thus their probability of participating in future interactions. This closes the feedback loop: persistence shapes the composition, composition biases the interaction, and the interaction generates new patterns whose persistence further reshapes the population.

A simple example illustrates the mechanism. Consider a system with base elements *A* and *B*, where the compound *AB* has a lifetime of 10 generations, *ABAB* has a lifetime of 50 generations, and all other compounds degrade instantly. After the first generation, only *A*, *B*, and *AB* persist; unstable combinations such as *AA* and *BB* are eliminated immediately. As more instances of *AB* accumulate due to its longer life, its frequency in the population increases, raising the probability that two copies of *AB* will be sampled together and recombined to form *ABAB*. Once present, *ABAB* persists five times longer than *AB*, accumulating further and biasing subsequent interactions toward still higher-order configurations. No fitness function was specified; no selection operator was imposed. The population-level bias toward *ABAB* emerged entirely from the differential between persistence times: stability created selection.

**Algorithm 1:**
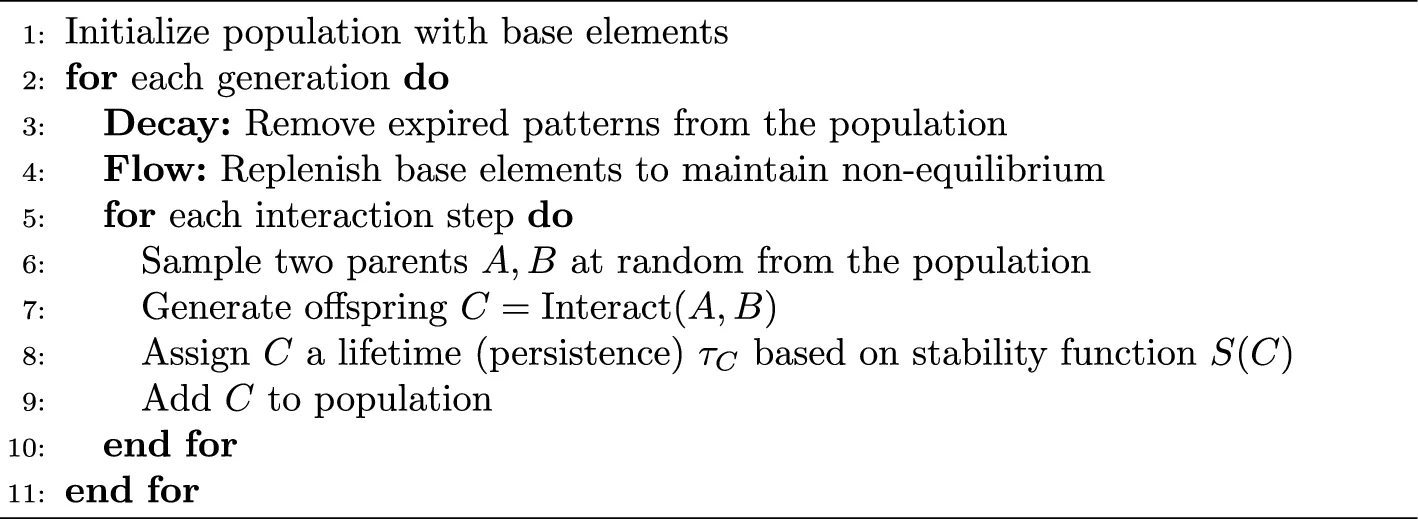
Stability-driven assembly (SDA): minimal constructive loop

Algorithm 1 formalizes the core loop. At each generation, patterns whose lifetimes have elapsed are removed, and base elements are replenished, maintaining the system far from equilibrium. Pairs of patterns are then drawn at random from the population. Because more persistent patterns have accumulated more instances, this uniform draw samples them more often, in proportion to their abundance, realizing roulette-wheel selection (Holland 1975) without any fitness criterion. The sampling rule is unbiased, and the bias toward stable structures enters only through the abundances that persistence has already shaped. The selected pair is combined to produce an offspring, and the offspring is assigned a lifetime determined by its stability. The population-level bias toward increasingly stable structures thus emerges from the asymmetry between creation and decay alone, with no fitness function specified.

This generative loop is similar to the canonical reactor dynamics of *artificial chemistries* (Dittrich et al. 2001), with one key difference: where they remove reactants and replenish the population uniformly, Algorithm 1 assigns each offspring a lifetime set by its stability, so that survival is differential rather than uniform. It is this single asymmetry, rather than any imposed fitness, that yields directional selection.

The stability function *S* is not an arbitrary parameter, but a physical property. The lifetime of a configuration is determined by its binding energies, decay rates, and structural reinforcement, always relative to a given environment. The same structure may be fleeting in one setting and long-lived in another. Across physics and chemistry these lifetimes span more than 30 orders of magnitude, from transient quantum states to structures that persist on geological or cosmological timescales.

In fact, it is the *equal* persistence case which is a fine-tuned exception. Uniformity would require lifetimes to coincide or correlate closely across structurally distinct configurations, whereas thermodynamic, kinetic, and structural differences generically produce differential persistence. These disparities are not incidental: they arise from symmetry, energetic minima, spatial organization, and hierarchical assembly, and they are kinetic rather than merely thermodynamic. For example, both an *ester* and a *peptide bond* will eventually break apart in water, yet the ester hydrolyzes within hours while the peptide bond survives for years without a catalyst. Consequently, even weak persistence biases, integrated over time and coupled to continual stochastic assembly, come to dominate population statistics. The SDA mechanism exploits an asymmetry that is the rule rather than the exception.

**Fig. 3:**
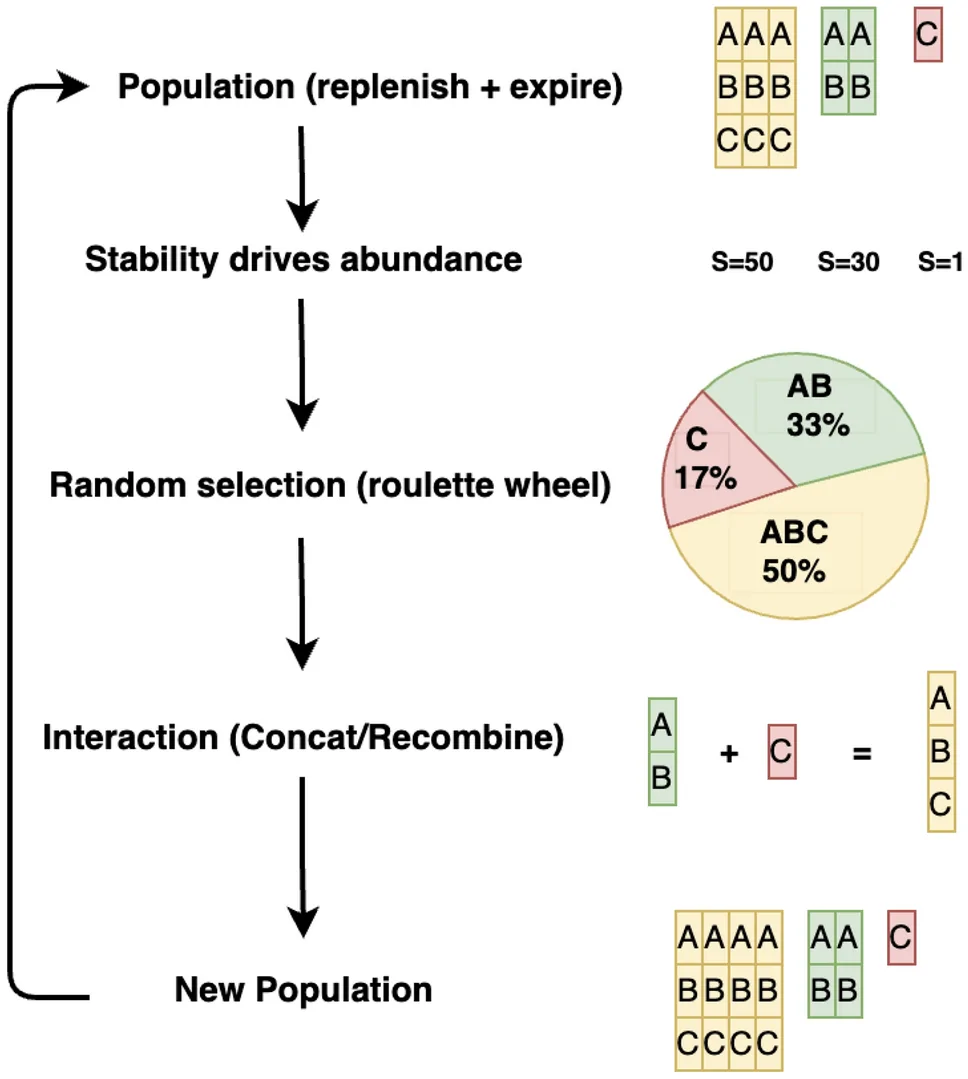
The constructive loop. Base elements are continuously replenished while unstable motifs expire. Patterns are sampled from the population, combined, and re-enter with lifetimes determined by their stability. The resulting feedback from stability to persistence to population composition produces emergent selection without externally specified fitness

As shown in Fig. 3, stability asymmetry leads to selection without the need for reproduction. Under replication, a pattern becomes abundant by direct lineage: each copy descends from common ancestors. Under SDA, the same pattern becomes abundant because it forms repeatedly from its components and persists once formed; the instances share no lineage, yet the standing population is identical. Selection acts on that standing population and is indifferent to the route that produced it. In either case, the population abundances set the roulette-wheel sampling probabilities.

To understand why this system breaks out of the *episodic cage effect* (Sect. “Episodic regimes as methodological artifacts”), it is useful to approximate population dynamics as a continuous transport process, by analogy with Kimura’s diffusion treatment of population genetics (Kimura 1964). In the limit of large populations, the evolution of the pattern density *P*(*x*, *t*) over a feature space *x* can be described by a Fokker–Planck equation (Gardiner 2009):

1 $$\begin{aligned} \frac{\partial P(x,t)}{\partial t} = \underbrace{- \nabla \cdot \big [ A[P](x,t)\, P(x,t) \big ]}_{\textit{Selection (Drift)}} \;+\; \underbrace{D \nabla ^2 P(x,t)}_{\textit{Variation (Diffusion)}} \end{aligned}$$

The Fokker–Planck equation decomposes the dynamics into two competing tendencies with opposite signs: a drift that transports probability mass toward favored regions of the space, and a diffusion that spreads it out towards uniformity. In Eq. 1, the diffusion term $D\nabla ^2 P$ captures the dispersive effect of stochastic pattern creation (exploration), while the drift term containing *A*[*P*] captures the population biasing effect of differential persistence (exploitation).

The crucial feature of Eq. 1 is the notation *A*[*P*]. In standard physical systems (e.g., a particle in a magnetic field), the drift field *A*(*x*) is an *external potential* fixed by the boundary conditions. It is an independent operator. However, in SDA, the drift is a *functional of the population distribution*. The “force” pulling the system toward specific regions of pattern space is not pre-existing; it is generated by the current population of stable scaffolds acting as attractors for future binding events.

This functional feedback (*A* depends on *P*, which later depends on *A*) is the mathematical signature of a constructive regime. The system is historically closed: the effective laws of motion at time *t* are determined by the accumulated structures from time *t-1*. This generally precludes the existence of a static equilibrium distribution that can be solved analytically (as in the Boltzmann distribution). To know the future distribution, one must integrate the path-dependent history of the functional *A*[*P*].

**Fig. 4:**
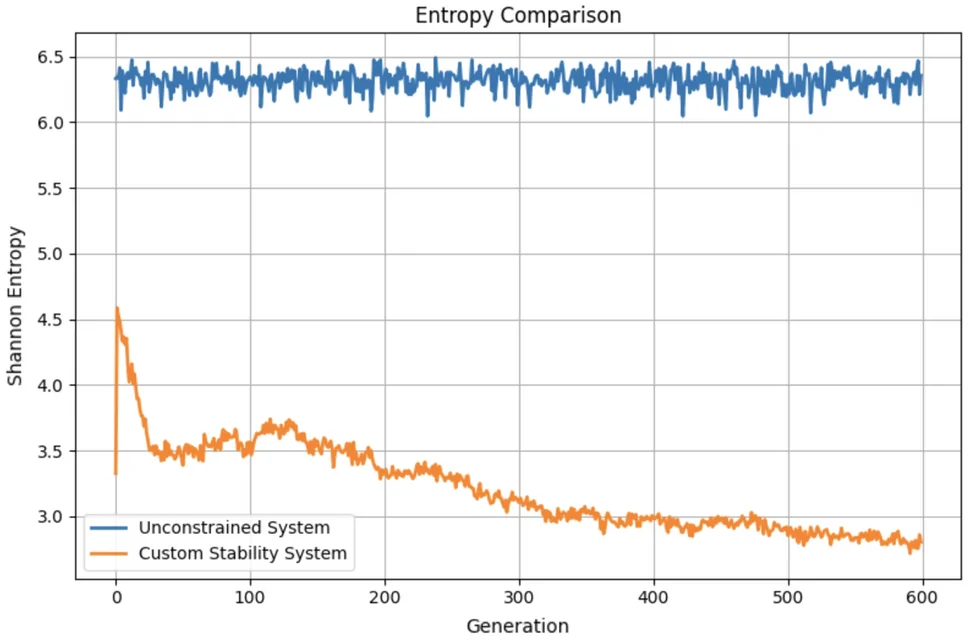
Entropy reduction via persistence. Evolution of the Shannon entropy of the population distribution in a symbolic SDA simulation (Adler 2026). In the unconstrained control (blue), entropy remains high as the system explores pattern space randomly. Under SDA dynamics (orange), entropy decreases as probability mass concentrates on families of stable, long-lived motifs

The consequence of this functional feedback is the spontaneous reduction of entropy. Figure 4 compares the Shannon entropy of a population under SDA dynamics against a neutral control. In the control case (random assembly without persistence bias), the system diffuses broadly through the combinatorial library (a regime we will later formalize as quadrant Q3), resulting in high entropy. In the SDA case, the population effectively discovers stable sub-structures. These structures persist, creating local density spikes that bias future sampling, leading to a cascading collapse of entropy as the system locks onto a set of dominant, stable scaffolds.

This result demonstrates that evolutionary ordering is not a unique property of biological replication. It may be a general physical property of open systems where persistence depends on structure. By simply allowing the output of an interaction to influence the probability of future interactions, SDA transitions the system from a fixed episodic probability distribution to an evolving constructive history.

The drift term of Eq. 1 plays a role analogous to the selection term of the Price equation (Price 1970), which relates the change in a population’s mean trait to the covariance between fitness and that trait. Here persistence plays the role of fitness, so a nonzero covariance arises only when persistence is differential. Crucially, the correspondence is diagnostic rather than generative: because the covariance must be recomputed in each generation as the population changes, it indicates that evolution is in progress, much like the entropy reduction of Fig. 4, but plays no role in producing the dynamics. The generative content lies in the functional feedback dependence of the drift *A*[*P*] on the population itself.

### From persistence to selection

**Fig. 5:**
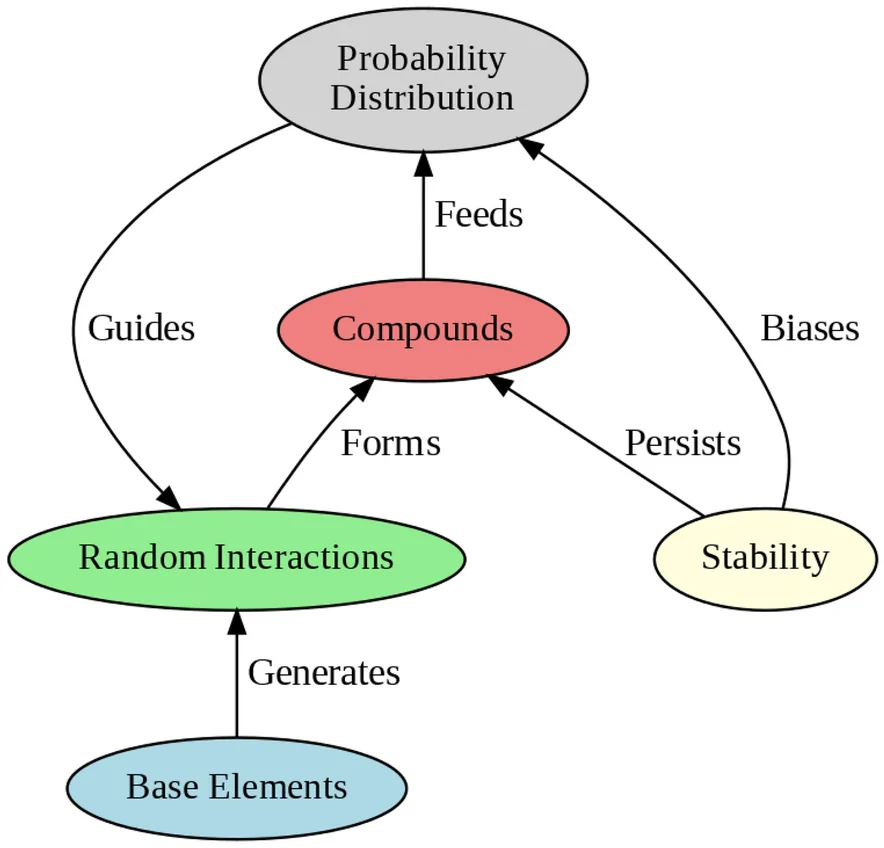
Core feedback structure underlying SDA. Differential persistence biases population composition, which biases future interactions, closing a causal loop in which selection emerges without genes, replication, or an imposed fitness function

Because sampling probability is proportional to population frequency, and frequency is determined by persistence, stability implicitly defines a fitness landscape. This feedback implements fitness-proportional (roulette-wheel) sampling (Goldberg 1989) without replication or an external fitness function. The system realizes a *natural genetic algorithm* (SDA/GA) (Adler 2026), in which variation enters through stochastic assembly and selection emerges from differential lifetimes alone. Figure 5 summarizes this feedback loop: persistence shapes population composition, composition biases future interactions, and interactions generate new patterns whose persistence further reshapes the population.

This feedback loop locates SDA within the debate over whether natural selection is a population-level causal process (Millstein 2006) or merely a statistical summary of individual births and deaths (Walsh et al. 2002). The downward arrow in Fig. 5, the probability distribution *guiding* the interactions that produce the next generation, is a genuine population-level cause. The distribution biases which interactions are more likely to occur, resulting in the roulette-wheel sampling characteristic of genetic algorithms (Holland 1975). This is causalist in a specific sense: the bias is produced by the feedback between bottom-up assembly and the erasure of configurations that fail to persist, and it is this loop, not any standing property of the population, that does the causal work of selection.

It is important to be precise about what “selection” means here. Since the Modern Synthesis, evolution has been understood through Lewontin’s triad: variation, differential fitness, and heredity (Lewontin 1970; Godfrey-Smith 2009). In practice, heredity is almost always operationalized as template copying: a mechanism by which individuals transmit information to offspring. This identification has become sufficiently entrenched that evolutionary dynamics, a population-level phenomenon, are often treated as inseparable from reproduction, an individual-level mechanism.

SDA satisfies the three conditions through different means. *Variation* arises from stochastic assembly. *Selection* arises from differential decay. *Heredity* arises not from template copying or replication, but from what we call *structural recurrence*: a persistent pattern remains available as a reactant, increasing the frequency of interactions that regenerate it, and more persistent structures containing it. The “offspring” of a pattern are new instances independently generated by the system’s interaction dynamics, sharing structural identity without sharing lineage, as shown in Fig. 3.

This generalization engages directly with Griesemer’s critique of the Modern Synthesis’s conflation of replication with reproduction (Griesemer 2000, 2005). Griesemer argues that heredity requires *material overlap* between generations: progenitor and offspring must share physical stuff for inheritance to occur. Structural recurrence is weaker still, requiring neither material continuity nor a copying mechanism, yet it remains sufficient to couple episodes into evolutionary trajectories.

As Maynard Smith argued (Maynard Smith and Szathmáry 1999), the earliest evolutionary systems likely operated under limited heredity without high-fidelity replication. Replication, on this view, is one particularly efficient implementation of structural recurrence, not the only one. Treating persistence rather than replication as the primitive selective quantity recasts evolution as a statistical property of population histories rather than a biological property of individuals, and extends evolutionary explanation to domains where replication is absent or undefined: prebiotic chemistry, institutional change, and the constructive regimes that are the subject of this paper. In the formalism introduced below, these interaction pathways correspond to instantiated *λ*-terms: each episode is a *λ*-application, and structural recurrence preserves *λ*-identity across independently generated instances.

Mitchell (Mitchell 2003) argues that biological phenomena require *integrative pluralism*: explanations that combine multiple levels and causal factors without expecting a reduction to a single framework. The SDA framework identifies a structural reason why: populations generate level-specific constraints, with the “rules” at each level of the explanatory hierarchy (Fig. 2) emerging from persistence-weighted dynamics at the level below. Mitchell’s pluralism thus finds a dynamical grounding in the recursive feedback structure of constructive systems.

This perspective also sharpens the debate over the Extended Evolutionary Synthesis (EES). Proponents argue that developmental plasticity, niche construction, and extra-genetic inheritance require expanding the neo-Darwinian account of evolutionary mechanism (Laland et al. 2015; Pigliucci and Müller 2010); critics respond that these phenomena are already accommodated within it (Wray et al. 2014). This dispute is about the *mechanism* of evolution. Our analysis bears on a prior question that both sides leave untouched: what makes a system evolutionary in the first place. SDA addresses that question, locating the answer in the conditions, differential persistence and population feedback, that distinguish a constructive regime from an episodic one.

### Turing versus Church: library versus lego

The equivalence of Turing machines and the *λ*-calculus is a cornerstone of computability theory: both capture the same class of effectively computable functions and confront the same undecidable boundaries (Turing 1936; Church 1936). Yet they achieve this equivalence through radically different architectures, and these differences matter when we move from the theory of *what can be computed* to the question of how computational structures *evolve*.

A Turing machine solves exactly one problem. It is episodic. Its state-transition table is fixed at design time; if you need a new function, you build a new machine. Each machine is like a *book in a library*: complete, static, and self-contained. Einstein captured this intuition: “We are in the position of a little child entering a huge library filled with books in many languages... The child dimly suspects a mysterious order in the arrangement of the books but doesn’t know what it is” (Isaacson 2007). In the episodic regime, the library is the correct metaphor: the book of nature is already written, and discovery is retrieval. But to expand the collection, one must write a new volume from scratch. There is no methodological reuse of internal structure across books except by deliberate offline copying.

The *λ*-calculus, in contrast, is compositional by nature. A *λ*-term is not a book but a *Lego block*: it can be applied to other terms, partially evaluated, passed as an argument, or returned as a result. New functions are assembled by applying existing terms to each other. The Church numeral $(\lambda f.\lambda x. f(fx))$ does not need to know in advance what *f* will be. It is a *scaffold* that awaits completion.

One might object that Von Neumann’s universal constructor shows a Turing machine *can* replicate itself (von Neumann 1966). But the replication is total and literal: the descendant is an identical copy, and the constructor must be built into the original design. There is no mechanism by which a machine, solving its own problem, spontaneously gives rise to a new machine solving a different problem. Innovation requires an a-priori architect. In the *λ*-calculus, composition is not a separate capability installed alongside a computation. It *is* itself a computation. A term that transforms *A* into *B* can be combined with a term that transforms *B* into *C*, producing a new term without rewriting either predecessor. The population remembers solutions not as discrete artifacts, but as recombinable capabilities. The library catalogs the past; the Lego set builds the future.

This is why the operators in Fig. 2 are written as *λ*-terms, not Turing tables: $\lambda _2$ arises from the population dynamics of $\lambda _1$ interactions because composition is already what $\lambda _1$ *does*. The machinery of emergence need not be installed from without; it is already operative within.

### Formalizing SDA in the *λ*-calculus

This structural equivalence was first recognized by Fontana (Fontana and Buss 1994), who mapped chemical objects to *λ*-calculus terms, precisely because the *λ*-calculus makes no distinction between program and data: every term is simultaneously a potential operator and a potential operand. Fontana showed that this reflects the ontological structure of constructive chemistry, where a molecule is both a thing acted upon (a reactant) and a thing that acts (a catalyst or scaffold). Fontana’s brilliant insight generalizes beyond chemistry: a firm is both an entity in a market and a set of procedures that transform inputs; a string is both a pattern to be recombined and a component that biases future recombination. The *λ*-calculus captures this shared constructive structure not because these domains are “really” computational, but because they are all systems in which the products of interaction re-enter the space of operators.

What SDA adds to Fontana’s ontological insight is the selection mechanism, which makes ths system evolve. Where Fontana required self-maintenance to filter the space of possible organizations, SDA shows that differential persistence alone is sufficient. Each recombination event in Algorithm 1 corresponds to a *λ*-application: two terms are drawn from the population, one acts upon the other, and the result either persists into $P_{t+1}$ or dissipates. The population is thus not a vector in a fixed state space but an evolving multiset of *λ*-terms whose composition at each generation determines the operators available for the next.

### Self-reference and the failure of spectral decomposition

In episodic regimes, the transition rules governing a system are time-invariant. A fixed linear operator can be spectrally decomposed into eigenvalues and eigenvectors, yielding closed-form expressions for long-run behavior. This is why physics admits symmetry arguments, dimensional reduction, and predictive shortcuts.

In SDA, this factorization is not generally available, and the reason is structural. The interaction rules at generation *t* depend on the population distribution $P_t$: which patterns are present, how abundant they are, and therefore which *λ*-applications are likely to occur. Since $P_t$ is itself the cumulative result of all prior interactions, the effective dynamics at each step encode the population history that produced them. To factor an operator from its operands, one must be able to specify the operator independently of the states upon which it acts. In SDA, the two are intertwined: the interaction rules cannot be written down without knowing $P_t$, and $P_t$ cannot be computed without evaluating the interaction rules in every preceding step.

Kleene’s recursion theorem offers a useful analogy: in a system able to represent its own operations, the rule applied at each step can depend on the system’s own state rather than being fixed in advance (Kleene 1938; Rogers 1987). SDA exhibits the same circularity. As established above, the interaction rule at generation *t* is a functional of the population $P_t$, which is itself the cumulative product of that rule applied at every prior step. Operator and operands are therefore inseparable, and an operator that cannot be specified independently of its operands admits no spectral decomposition: there is no fixed matrix to diagonalize, no time-independent Hamiltonian to solve. The dynamics are irreducibly sequential, and this *generative irreducibility* is the formal basis of the constraint developed in Section “The “no free telos” constraint”.

This does not mean that prediction is never possible. When the population distribution changes slowly, dominated by a few high-stability scaffolds, the effective dynamics may remain approximately fixed, and standard analytical tools may be applied locally. The system behaves, for a time, *as if* it were episodic. But this equilibrium is always provisional: the same persistence-weighted feedback that stabilizes the current configuration also concentrates probability mass on the interactions most likely to produce novel high-stability compounds. When such a compound emerges, it can reorganize the entire population distribution, invalidating the local approximation. Predictability in constructive systems is thus intermittent: present during quasi-stable periods, absent across the transitions between them.

## The four quadrants

**Fig. 6:**
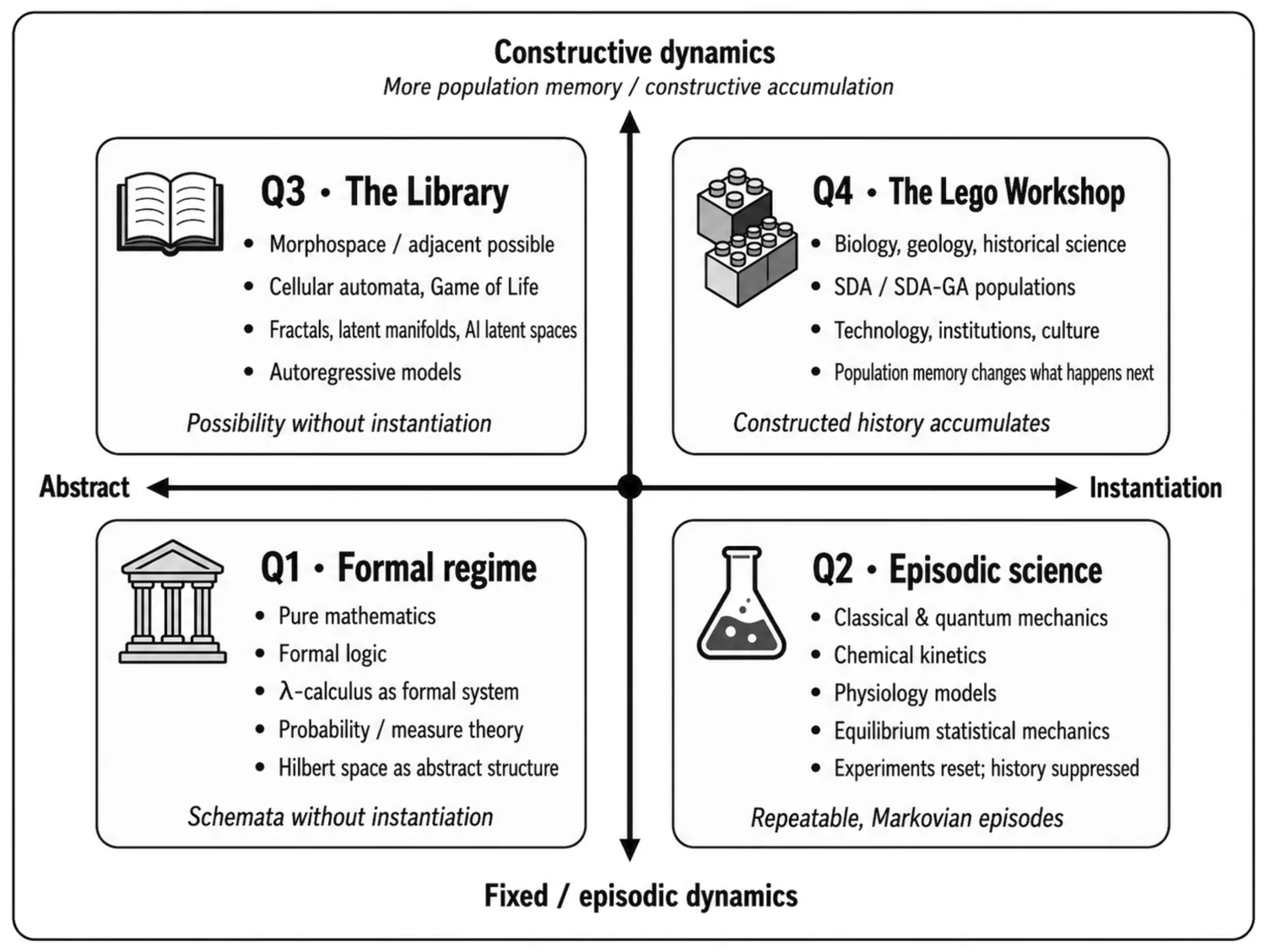
The four quadrants: The horizontal axis distinguishes abstract formal schemata from physically instantiated populations. The vertical axis distinguishes fixed dynamics (invariant operators on fixed state spaces) from constructive dynamics (operators and state spaces co-evolve)

The preceding analysis suggests that the distinction between episodic and evolutionary explanation is not a matter of domain but of dynamical structure. We organize scientific explanation along two independent dimensions: whether the objects of study are abstract formal schemata or physically instantiated populations, and whether the governing dynamics are fixed or endogenously modified by the process itself. The resulting map (Fig. 6) locates episodic science, evolutionary science, and two commonly conflated intermediate cases within a single framework.

*Q1: The formal regime* (abstract, fixed). Pure mathematics, formal logic, and the *λ*-calculus *as a formal system* occupy this quadrant. Objects are purely abstract, not instantiated in any physical reality, but rather defined by axioms and inference rules that do not change. A theorem proved today was provable yesterday and will be provable tomorrow. Q1 provides the schemata and the *λ*-terms that other quadrants instantiate, but it contains no populations, no time, and no history. Whether these formal structures exist independently of human cognition (as mathematical Platonism holds) or are epistemic constructions is not debated here. What matters for our framework is that they place inviolable constraints on the dynamics of the quadrants in which they are instantiated.

*Q2: Episodic science* (instantiated, fixed). This is the home of classical and quantum mechanics, chemical kinetics, physiology, and equilibrium statistical mechanics, systems where physically realized populations exist but episodes are independent. The apparatus is reset between trials, the state space is fixed in advance, and the transition rules are invariant. Prediction succeeds here because the operator *F* in $x_{t+1} = F(x_t)$ can be specified independently of the states upon which it acts, allowing spectral decomposition, and all the predictive machinery of Section “Self-reference and the failure of spectral decomposition”. Classical game theory and mean-field economics also reside here: agents interact within a fixed strategy space, and selection operates over states rather than structures. Q2 is where Wigner’s “unreasonable effectiveness” holds, precisely because Methodological Markovianity is enforced.

*Q3: The library* (abstract, constructive morphospace). This quadrant contains possibility spaces, such as the space of all possible proteins or the adjacent possible (Kauffman 2019) taken as a static set, whose structure is combinatorially rich and, in principle, open-ended, but which contain no physically instantiated populations. Iterative and recursive systems such as cellular automata (Wolfram 2002), Conway’s Game of Life, and fractal generators also reside here. They can exhibit complex, seemingly evolutionary behavior, but their dynamics unfold under fixed rules with no population-level feedback and no instantiation in the physical world. Because such systems can be recursive and even self-referential, generating elaborate structure from simple rules, it is sometimes tempting to treat them as the hidden generative engine of Q4. But recursion within a fixed rule set is not construction: they enumerate possibilities within the cage of their initial conditions, never expanding the space they explore. Autoregressive models and systems that map to and from fixed latent spaces similarly inhabit Q3. They may carry memory of their past, but lack population-level selection over physically instantiated variants. Q3 answers *what could exist*, not *what does exist*: it catalogs the Library but does not write new books.

*Q4: The lego workshop* (instantiated, constructive). Biology, geology, real (non-equilibrium) economics, technology, and cultural evolution occupy this quadrant. Here, populations of physically realized structures persist differentially, they feed back on interaction probabilities, and generate novel structures that re-enter the space of operators. The effective dynamics at each step are shaped by the population that previous steps produced. Q4 is where the *λ*-schemata of Q1, constrained by the laws discovered in Q2, are instantiated as evolving populations whose trajectories cannot be predicted without simulation.

The quadrants are not hermetic. Movement between them defines the central transitions in the history of science. The passage from Q1 to Q2 is *instantiation*: a formal schema acquires physical referents and becomes an empirical law. Newton’s inverse-square law exists as a mathematical relation in Q1; applied to planetary orbits, it becomes episodic science in Q2. The passage from Q2 to Q4 is the main claim of the paper: episodic science becomes evolutionary science when population-level memory couples episodes over time. A chemical reaction studied in isolation in Q2, becomes a constructive system when its products persist and bias future reactions in Q4.

The passage from Q3 to Q4 is *actualization*. The Library contains every possible protein; the Workshop contains only those that a specific history of persistence-weighted assembly has constructed. No amount of contemplation of Q3 produces a single entry in Q4. For that, one needs energy, time, and a population. The distinction between Q3 and Q4 echoes Kant’s observation (Kant 1781) that one cannot reason one’s way from a concept to an object. The concept of one hundred dollars is identical to the concept of one hundred *existing* dollars, yet the financial consequences are quite different. The *possibility* of a complex organism is a mathematical fact given by the laws of chemistry and combinatorics, but its *actuality* requires a constructive history.

The distinction between Q2 and Q4 reframes the long-standing divide between sciences that seek general laws and those that reconstruct particular histories (Windelband 1894; Beatty 1993). What determines whether a science is historical is not its subject matter (e.g. chemistry studies repeatable reactions, yet geochemistry is historical), but whether its explanatory framework structurally permits or suppresses population-level memory.

Kauffman’s concept of the adjacent possible (Kauffman 2019) can be understood as the dynamic boundary between Q3 and Q4. For any instantiated population in the Lego Workshop, the adjacent possible constitutes the immediate subset of the Library that is exactly one constructive step away from realization. As the population actualizes novel structures through persistence-biased interaction, the boundary expands into previously inaccessible regions of Q3. Because the adjacent possible of tomorrow depends entirely on what is constructed today, the trajectory through the Library cannot be mapped in advance; it must be built.

Relational approaches such as Rosen (1991) correctly identify Q4 as the target regime by emphasizing organizational closure. However, by treating closure as a definitional primitive rather than an emergent outcome of physical dynamics, they leave the constructive mechanism unspecified: how biological closure is achieved from non-living precursors.

The closure of constraints framework of Mossio et al. (2013); Moreno and Mossio (2015) substantially refines this position, grounding closure in the emergent causal powers of constraints and the thermodynamic flow they channel. Closure is defined there as collective self-maintenance: a network of constraints, each conserved at its relevant timescale, whose maintenance conditions are mutually produced by the others. This presupposes constraints that already persist differentially on different timescales. The closure relation organizes such persistent structures into a self-maintaining whole, but does not generate their persistence. SDA addresses this prior question, identifying differential persistence as a generative mechanism that determines which configurations endure long enough to act as constraints in the first place.

## Thermodynamic erasure and the cost of construction

The Kantian gap into Q4 is not only epistemic but also thermodynamic. In the episodic regime, dynamics are fundamentally reversible: unitary quantum evolution and Hamiltonian classical mechanics preserve information. Given the final state, one can, in principle, recover the initial state. Evolutionary construction is irreversible because selection is information erasure: to construct a specific structure from a vast combinatorial space requires discarding the configurations that were *not* selected.

This connection is formalized by Landauer’s Principle (Landauer 1961): erasing a single bit of information requires dissipating at least $k_B T \ln 2$ of heat into the environment. In SDA, every decay event is a Landauer erasure: the system forgets a failed configuration. When the population converges on a stable scaffold, it reduces the entropy of the probability distribution (Fig. 4), and this reduction must be paid for by exporting entropy to the environment. The population acts as if it were a collective “Maxwell’s Demon" (Bennett 1982). Interaction tests for stability. Decay erases what fails. Energy flow pays the bill, flushing the entropy of rejected configurations into the environment so that the survivors can accumulate and compound.

## The “no free telos” constraint

### Formal statement and scope

The preceding analysis yields a constraint on prediction in constructive systems. The generative irreducibility identified in Section “Self-reference and the failure of spectral decomposition” (the interdependence of operator and population) means that no general algorithm can compress the constructive history into a predictive shortcut.

Turing’s undecidability results (Turing 1936) establish that no *general* algorithm can predict the outcome of an arbitrary self-referential computational process. SDA systems, in which the interaction operator is a functional of the population history, exhibit the self-referential structure to which these results apply. Whether they are Turing-complete in the strict sense remains an open formal question, but the dynamical mechanism where operators rewrite the conditions for their own future application produces a halting-problem-like obstruction to universal shortcut prediction. This does not mean that every constructive system resists prediction at all times. Just as specific programs can be proven to halt even though the halting problem is undecidable in general, specific evolutionary trajectories may admit local prediction even though the class of constructive systems does not admit a universal predictive shortcut. What the formal results *do* establish is that any such predictability is *fragile* and vulnerable to disruption by emergent structure.

**Fig. 7:**
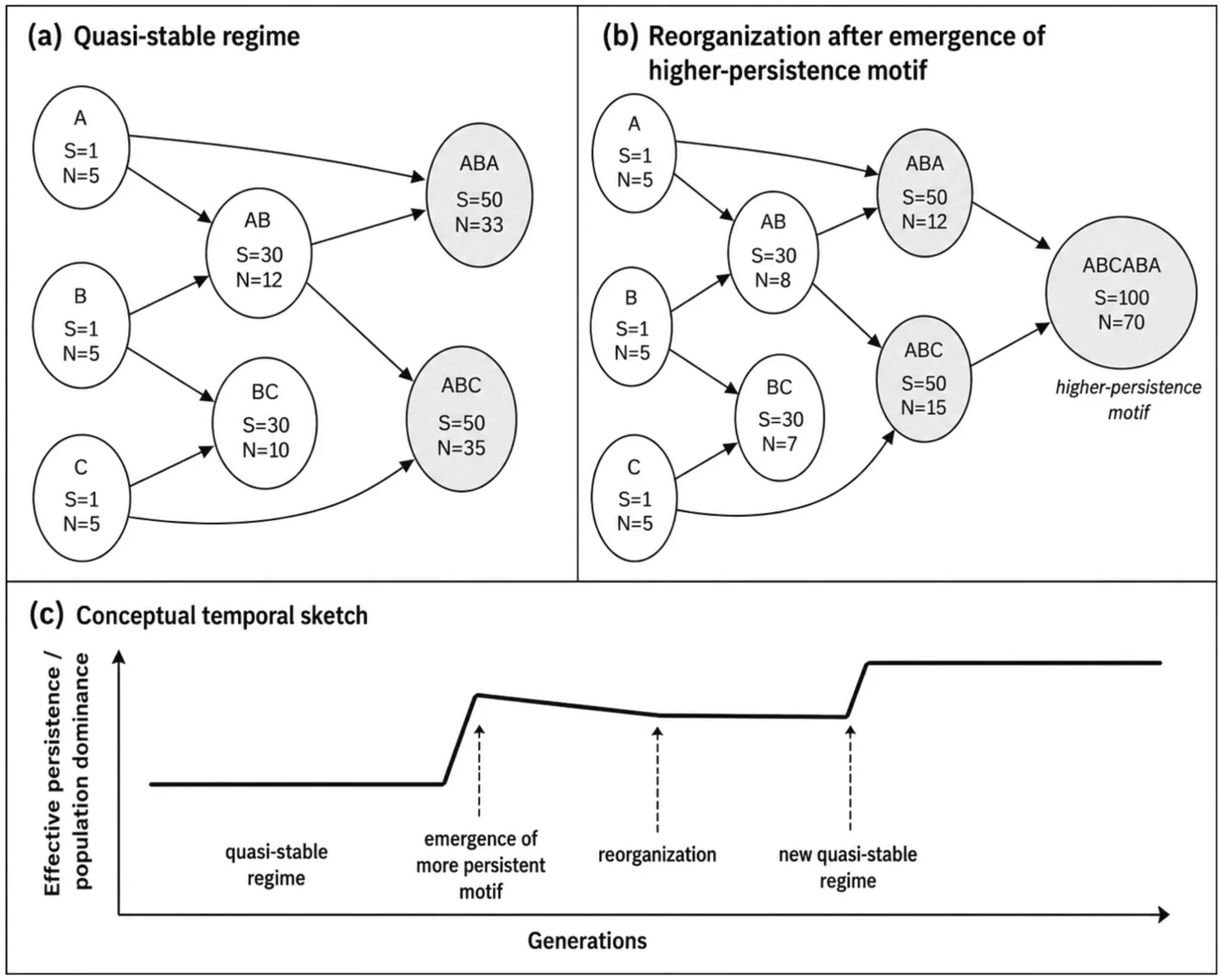
Punctuated dynamics in SDA. **a** A quasi-stable regime in which ABA and ABC dominate the population. **b** A later generation after the emergence of the higher-persistence motif ABCABA (*S=100*), which redirects probability mass flow and produces a new dominant configuration. **c** Conceptual temporal sketch: apparent stability is interrupted by the emergence of more persistent motifs, triggering reorganization to a new quasi-stable regime. Here *S* denotes pattern stability, measured as characteristic lifetime in generations, and *N* denotes pattern instance count

Figure 7 illustrates how constructive systems can exhibit punctuated dynamics. The transition from the quasi-stable configuration in Fig. 7a to the reorganized configuration in Fig. 7b occurs when ABCABA emerges from the interaction of ABC instances with ABA instances. Because this interaction depends on the prior abundance of ABC and ABA, its probability is historically conditioned by the existing population state. Once ABCABA starts to appear due to stochastic interactions, its higher persistence changes the sampling distribution itself, redirecting probability mass flow, and altering the system’s effective dynamics. The resulting pattern is not gradual relaxation toward a fixed equilibrium, but stasis interrupted by reorganization. This provides a dynamical interpretation of punctuation in constructive domains, including punctuated equilibrium in paleontology (Eldredge and Gould 1972), creative destruction in economics (Schumpeter 1942), and paradigm shifts in the history of science (Kuhn 1962). The SDA framework explains why such punctuation can arise generically: stable scaffolds concentrate probability mass, increasing the likelihood of interactions that generate novel combinations with still higher persistence. In this sense, stasis creates the conditions for its own punctuation.

#### Constraint 1

(No Free Telos - NFT) For a constructive system in which the effective interaction rules at generation *t* depend functionally on the population history $P_0 \ldots P_t :$

1. *Local prediction* is possible during quasi-stable periods when the population distribution changes slowly relative to the observation timescale.
2. *Global prediction* determining the dominant structures at time *T ≫ t* requires running the generative history. The emergence of disruptor structures cannot be anticipated without computing the interaction sequence that produces them.
3. *Transitions* between quasi-stable regimes are generically unpredictable: perturbations of all sizes, including system-reorganizing cascades, occur with nonzero probability.

The claim is not that prediction is impossible, but that predictability is *intermittent*. The telos is not free because the cost of crossing the Kantian gap, from possibility (the space of potential disruptors) to actuality (which disruptor emerges and when), is paid in computational steps that cannot be compressed.

### The assembly index as empirical indicator

If constructive history is physically encoded in structure, we should expect a measurable signature. Assembly Theory (Sharma et al. 2023) offers one such metric. The Assembly Index ($M_A$) of an object is the minimum number of recursive join operations required to construct it from basic building blocks. In our framework, $M_A$ may serve as a lower-bound metric for the depth of the constructive path.

A crucial distinction must be drawn between $M_A$ and the actual trajectory. The Assembly Index calculates the *shortest* construction path. It is a compression metric. But SDA evolution selects for persistence, not parsimony. As Koza demonstrated in his work on *Genetic Programming* (Koza 1992), evolved solutions rarely converge to minimal implementations. They exhibit “bloat” and redundant structures carried along by the process, unless parsimony is explicitly included in the fitness function, which is rarely the case in natural evolution. The population finds *a* path, not necessarily the shortest path. Thus, $M_A$ represents the theoretical minimum cost of construction. The actual cost is likely much higher.

## Conclusion

The success of the physical sciences has long encouraged the hope that if we could find the fundamental laws and the initial conditions, the structure of the complex world would follow as a necessary consequence. This is the dream of the Library: the book of the universe is already written, and science is learning to read it.

We have argued that this dream rests on a regime-defining idealization. The methods of episodic science: spectral decomposition, fixed state spaces, and invariant operators are effective precisely because they are restricted to systems where population-level memory is structurally suppressed. By imposing Methodological Markovianity, we filter out the mechanism that makes evolution possible: the feedback by which persistent structures reshape the interactions that produce them.

The shift to a constructive epistemology does not abandon episodic science, but extends it. The four-quadrant map developed here sharpens a persistent tension in the philosophy of science: the apparent divide between law-based and historical explanation reflects neither a hierarchy of rigor nor an accident of subject matter, but a structural difference in epistemic regime. Physics and biology are not in conflict; they occupy different quadrants of the same map. The domain-specific *λ*-schemata discoverable by episodic science define the constraints, the geometry of the studs, but the population trajectory through the space of possible constructions may be determined by persistence-weighted feedback that rewrites the effective dynamics at every step to build a specific castle. The Library (Q3) catalogues what is possible; the Lego Workshop (Q4) constructs what is actual. The “No Free Telos” constraint, grounded in the self-referential structure of constructive dynamics and Turing’s undecidability results (Turing 1936), establishes that no general shortcut bridges the gap between them.

A recurring impulse in the history of thought, from Platonic forms to Pythagorean harmonics, to contemporary proposals that identify physical reality with the space of possible computations (Wolfram 2021), has been to locate causal priority in the space of possibility. In this view, the actual world is governed by, or derived from, an abstract order that precedes it. In the language of our framework, these accounts treat Q1–Q3 as causally prior to Q4. The SDA framework inverts this priority.

This inversion does not diminish the role of episodic science. The laws discoverable in Q1–Q3 are the *λ*-schemata that constrain every constructive step in Q4: conical symmetries give rise to inverse-square force laws, molecular orbital geometry determines which bonds are stable, and thermodynamic inequalities set the cost of each Landauer erasure. These constraints are real and inviolable. What they do not do is determine the trajectory. The geometry of the studs is fixed by physics; which castle gets built is determined by the population history of persistence-weighted assembly. SDA does not replace episodic science, but identifies what episodic science cannot, in principle, provide: an account of how particular constructions emerge from the space of possibilities that physical law permits.

The constructive order of the Lego Workshop is not derived from the Library; it is assembled within it, through stochastic interactions, differential persistence, and population-level feedback. It presupposes no blueprint and no external fitness function, only an open thermodynamic system with asymmetries in stability, conditions generic to any universe whose initial symmetry is broken. The natural genetic algorithm of the SDA/GA model (Adler 2026) already realizes this concretely, exhibiting the emergence of dominant scaffolds and sustained novelty in a chemical symbol space over thousands of generations without genes or imposed fitness.

The present SDA/GA framework establishes the epistemological status of constructive systems, but it is not a complete evolutionary theory. It does not address how stability functions themselves arise, how environmental boundary conditions change, or how the hierarchical organization of Fig. 2 bootstraps across levels. Its predictions are, moreover, currently demonstrated by symbolic simulation rather than empirical data; how differential persistence drives constructive dynamics in real prebiotic, geological, or economic systems remains an open empirical question. A natural next step is to extend the SDA/GA simulations toward these questions, in particular toward multi-level and environmentally coupled regimes, and to test the framework empirically in systems where stability can be measured directly.

The “No Free Telos” constraint establishes that global prediction in constructive systems is generally irreducible, but it does not preclude *local anticipation*. During quasi-stable periods, the current population distribution determines which *λ*-applications are probable. Where that distribution is observable, future work may aim to predict the next set of high-probability disruptor states. This would not predict *when* disruption occurs, but would narrow *what* it is likely to look like, turning the NFT constraint from a limitation on prediction into a guide for anticipatory modeling. Such an approach could ground empirical studies in prebiotic chemistry, where the relevant stability landscapes are increasingly measurable.

## Data Availability

No datasets were generated or analysed during the current study.
